# Data on physicochemical properties of active films derived from plantain flour/PCL blends developed under reactive extrusion conditions

**DOI:** 10.1016/j.dib.2017.09.071

**Published:** 2017-09-30

**Authors:** Tomy J. Gutiérrez, Vera A. Alvarez

**Affiliations:** Grupo de Materiales Compuestos Termoplásticos (CoMP), Instituto de Investigaciones en Ciencia y Tecnología de Materiales (INTEMA), Facultad de Ingeniería, Universidad Nacional de Mar del Plata (UNMdP) y Consejo Nacional de Investigaciones Científicas y Técnicas (CONICET), Colón 10850, B7608FLC Mar del Plata, Argentina

**Keywords:** Active films, Antimicrobial properties, Cross-linking, Poly(ε-caprolactone), Starchy

## Abstract

The data given below relates to the research paper entitled: “Eco-friendly films prepared from plantain flour/PCL blends under reactive extrusion conditions using zirconium octanoate as a catalyst”, recently published by our research group [1]. This article provides information concerning the physicochemical properties of the above-mentioned film systems: thickness, density, opacity, moisture content and surface moisture.

**Specifications Table**

**Table:** 

Subject area	*Polymers.*
More specific subject area	*Active eco-friendly films derived from plantain flour/PCL blends using zirconium octanoate (Zr(Oct)*_*4*_*) as a catalyst under reactive extrusion (REx) conditions.*
Type of data	*Table.*
How data was acquired	*Thickness was determined with a digital micrometer. Density and moisture content were calculated gravimetrically. Opacity and surface moisture were estimated with the aid of a UV–vis spectrophotometer (u-2001) and a Moisture Analyzer (Model MA150), respectively.*
Data format	*Raw, calculated and analyzed.*
Experimental factors	*The films were conditioned at ∼ 57% relative humidity in desiccators at 25 °C for 7 days using a saturated NaBr solution.*
Experimental features	*Film thickness was determined from eighteen random positions on each sample. Film density and moisture contents were determined at 105* ± *1 °C for 24 h. Film opacity was measured at 600 nm. Surface moisture content of the different films was measured after drying at 105 °C for 120 s.*
Data source location	*Mar del Plata, Argentina.*
Data accessibility	*Data are presented in this article.*

**Value of the data**

• The data gives detailed descriptions of the physicochemical properties of active eco-friendly films based on plantain flour/PCL blends using Zr(Oct)_4_ as a catalyst under REx conditions.
• The data provides information to researchers about the effects of the catalyst and the PCL-containing blends on the opacity of the materials.

## 1 Data

The data given in this study (Table 1) shows the physicochemical properties (thickness, density, opacity, moisture content and surface moisture) of active films derived from plantain flour/PCL blends under REx conditions, described in the article by Gutiérrez and Alvarez [1]. These characteristics add to the properties previously investigated [1]. This data widens the knowledge we have about the physicochemical properties of plantain flour/PCL systems cross-linked under REx conditions using Zr(Oct)_4_ as a catalyst. Thickness, density, opacity, moisture content as well as surface moisture were evaluated.

**Table 1:** Thickness (*e*), density (*ρ*), opacity, moisture content (*MC*) and surface moisture of the different films.

**Parameter**	TPPF	TPPF/PCL(10,000)	TPPF/PCL(10,000)+CAT	TPPF/PCL(80,000)	TPPF/PCL(80,000)+CAT
***e*****(mm)**	1.23 ± 0.04^b^	1.03 ± 0.04^a^	1.04 ± 0.04^a^	1.17 ± 0.06^b^	1.14 ± 0.05^b^
***ρ*****(g/cm**^**3**^**)**	1.2 ± 0.1^a^	1.24 ± 0.03^a^	1.19 ± 0.04^a^	1.1 ± 0.1^a^	1.16 ± 0.09^a^
**Opacity**	0.32 ± 0.01^d^	0.20 ± 0.01^b^	0.22 ± 0.01^b,c^	0.16 ± 0.01^a^	0.19 ± 0.01^b^
***MC*****(%)**	17.2 ± 0.5^b,c^	16.1 ± 0.6^b^	16.64 ± 0.08^b^	16 ± 1^b^	13.3 ± 0.4^a^
**Surface moisture (%)**	0.9 ± 0.6^a^	0.8 ± 0.2^a^	0.7 ± 0.1^a^	0.8 ± 0.1^a^	0.72 ± 0.02^a^

Equal letters in the same row indicate no statistically significant differences (*p* ≤ 0.05).

Film systems: plantain flour (TPPF), plantain flour + PCL (*M*_w_ = 10,000 g/mol) (TPPF/PCL(10,000)), plantain flour + PCL (*M*_w_ = 10,000 g/mol) + catalyst (TPPF/PCL(10,000)+CAT), plantain flour + PCL (*M*_w_ = 80,000 g/mol) (TPPF/PCL(80,000)) and plantain flour + PCL (*M*_w_ = 80,000 g/mol) + catalyst (TPPF/PCL(80,000)+CAT).

## 2 Experimental design, materials and methods

The systems characterized here are the active eco-friendly films that were studied in [1]. All the films were prepared from plantain flour/PCL blends under REx conditions using Zr(Oct)_4_ as a catalyst. The blends were then thermo-compressed to obtain the films. Five film systems were developed as follows: plantain flour (*TPPF*), plantain flour + PCL (*M*_w_ = 10,000 g/mol) (*TPPF/PCL(10,000)*), plantain flour + PCL (*M*_w_ = 10,000 g/mol) + catalyst (*TPPF/PCL(10,000)+CAT*), plantain flour + PCL (*M*_w_ = 80,000 g/mol) (*TPPF/PCL(80,000)*) and plantain flour + PCL (*M*_w_ = 80,000 g/mol) + catalyst (*TPPF/PCL(80,000)+CAT*). The resulting materials were conditioned with a saturated NaBr solution (*a*_*w*_ ∼0.575 at 25 °C) for seven days prior to each test.

### 2.1 Film characterization

#### 2.1.1 Thickness

Film thickness was determined using a digital micrometer (Micromaster^®^, Mitutoyo, USA) with 0.001 mm accuracy. Measurements were taken from eighteen random positions on each sample. Results were reported as the average thickness ± standard deviation (SD). These mean values were used to determine the density and opacity as described in the following sections.

#### 2.1.2 Density

Film density (*ρ*) was determined by cutting samples of each film type into 6 mm radius (*r*) discs. The thickness (*e*) of the discs was then determined by taking 18 random measurements of each one. Afterwards, the discs were dried at 105 °C for 24 h and weighed. The density was then calculated as the ratio between the weight and volume (thickness × area) of each disc using the equation proposed by Gutiérrez [2]:

(1) $$\rho =W_{i}-W_{f}/r^{2}*\Pi *e$$

where *W*_i_ is the initial dry weight, and *W*_f_ the final dry weight.

Results were reported as the density ± SD from three measurements.

#### 2.1.3 Opacity

The opacity was determined according to the method proposed by Sukhija et al. [3]. The ultraviolet (UV) and visible light barrier properties of dried films were measured at selected wavelengths between 400 and 800 nm using a UV–vis spectrophotometer (u-2001, Japan). Film opacity was measured at 600 nm and calculated using the following equation given by Han and Floros [4]:

(2) $$\mathrm{Opacity}=A_{600}/e$$

where: *A*_600_ = the absorbance at 600 nm and “*e*” = film thickness (mm).

#### 2.1.4 Moisture content (MC)

Moisture content of the films was calculated gravimetrically. For this, square-shaped samples (4 cm^2^) were weighed using an analytical balance (±0.0001 g; Ohaus, USA) in order to determine their initial mass (m_i_). The samples were then placed in an oven at 105 ± 1 °C (Memmert, Germany) for 24 h until constant weight (final dry mass = *m*_d_) was reached. All the tests were performed in triplicate, and the means ± SD were reported. Moisture content was determined by the following equation:

(3) $$\mathrm{MC}(\%)=\left(m_{i}-m_{d}/m_{i}\right)\times 100$$

#### 2.1.5 Surface moisture

The surface moisture contents of the films were determined using a Moisture Analyzer, Model MA150 (Goettingen, Germany). Square shaped samples (2 × 2 cm) were dried at 105 °C for 120 s. Measurements were then conducted in triplicate for each film and the results were reported as % of average surface moisture ± SD.
