# Supplementary material for: Data on physicochemical properties of active films derived from plantain flour/PCL blends developed under reactive extrusion conditions
Source: Data Brief. 2017 Sep 30;15:445–8. doi: 10.1016/j.dib.2017.09.071 (PMC5645474; doi:10.1016/j.dib.2017.09.071)
Supplement: Supplementary file 1 — Supplementary material [file mmc1.pdf]

Conflicts of interest

The authors declares no conflict of interest.
